# A new species of *Schinia* Hübner from the southeastern United States (Lepidoptera, Noctuidae, Heliothinae)

**DOI:** 10.3897/zookeys.52.476

**Published:** 2010-07-30

**Authors:** Michael G. Pogue

**Affiliations:** Systematic Entomology Laboratory, PSI, Agricultural Research Service, U. S. Department of Agriculture, c/o Smithsonian Institution, P.O. Box 37012, NMNH, MRC-168, Washington, DC 20013-7012, USA

**Keywords:** Southern coastal plain, East Gulf coastal plain, sand dunes, taxonomy

## Abstract

Schinia psamathea **sp. n.** is described from the southern coastal plain in Georgia and the East Gulf coastal plain in Florida and Alabama in habitats associated with sandy soil or dunes. Adult males and females and their genitalia are described and illustrated. Schinia psamathea is compared to Schinia saturata (Grote).

## Introduction

Since Hardwick (1996) revised the North American Heliothinae there have been several new species described in the genus Schinia Hübner (Knudson, Bordelon, and Pogue 2003; Pogue and Harp 2003; Pogue and Harp 2004; Pogue 2004; Pogue and Harp 2005). This paper describes a new species that seems to be associated with sandy areas and dunes of the southern coastal plain in Georgia and the East Gulf coastal plain in Alabama and Florida.

A revision of the Heliothinae is currently in preparation and the genus Schinia will be divided into numerous species groups based on morphology. This new species is in the gracilenta species group and will be included in a key to this group within the revision of Schinia in the Moths of North America fascicle on the Heliothinae.

The distribution of Schinia psamathea sp. n. includes coastal dune areas within the Bon Secour National Wildlife Refuge in southeastern Alabama and St. Joseph Peninsula State Park in Gulf Co., Florida. These areas are in potential danger of the British Petroleum oil spill in the Gulf of Mexico. Schinia psamathea sp. n. is being described in order to track any changes in its population dynamics along the Gulf Coast that may be due to this oil spill.

The solid brown color of the forewing, the absence of or faint antemedial and postmedial lines and the solid, slightly darker brown hind wing will separate Schinia psamathea sp. n. from Schinia saturata (Grote) in the southeastern United States.

## Methods

Genitalia dissections follow the method described in Pogue (2002) except specimens were mounted in Euparal and stained in Mercurochrome. Terms used in the descriptions of male genitalia follow Forbes (1954) and those for the female follow Klots (1970). Images of adult moths and genitalia were taken with a Visionary Digital Imaging System using a Nikon D1X camera with a modified K2 long-distance lens and a pulsed xenon flash. Forewing length was measured using a calibrated ocular micrometer from the juncture of the thorax to the apex, including fringe.

Specimens examined were from the private collection of Charles E. Harp, Littleton, CO (CEH), Mississippi Entomological Museum, Mississippi State University, Mississippi State University, MS (MEM), and the National Museum of Natural History, Smithsonian Institution, Washington, DC (USNM).

## Systematics

### Schinia psamathea

Pogue sp. n.

urn:lsid:zoobank.org:act:20E6AA9D-5468-4FEA-9E67-73C9CFCA5E35

Figs 1–4 9–12 

#### Type material.

Holotype ♂. USA, Alabama, Baldwin Co., 1 mi E Oyster Bay, T9S, R4E, Sec. 7 NW, 13 Oct. 1990, R.L. Brown, MEM 34951. Deposited in USNM. Paratypes: 153 ♂, 49 ♀: USA, Alabama. Baldwin Co., 56 ♂, same data as for holotype, ♂ genitalia slide USNM 51792; Bon Secour National Wildlife Refuge, T9S, R2E, Sec. 25 S, 5 Oct. 1996 (3 ♂), genitalia slide USNM 51384, J. Slotten, 12–16 Oct. 1991 (39 ♂, 32 ♀), R. Brown, D. Pollock, ♂ genitalia USNM 51794; Bon Secour National Wildlife Refuge, T9S, R3E, Sec. 11NW, 13–14 Oct. 1991 (1 ♂), R. Brown, D. Pollock; Bon Secour National Wildlife Refuge, 15 Oct. 1996 (36 ♂, 9 ♀) ♂ genitalia slide USNM 51793, T.L. Schiefer, 17 Oct. 1997 (3 ♂, 1 ♀), R.L. Brown; east of Mobile St., approximately 250 yds from beach, 21 Oct. 2000 (1 ♀), H. Grisham & R. Brown. Florida. Gulf Co., St. Joseph Peninsula State Park, 24 Oct. 2000 (1 ♂), J. Slotten. Okaloosa Co., Shalimar, 29 Sep. 1964 (1 ), 1 Oct. 1964 (1 ♂), 1 Oct. 1965 (1 ♂), 22 Oct. 1965 (1 ♂), H.O. Hilton. Walton Co., 0.5 mile S of I-10 on Rd. 285, 3 Oct. 2004 (5 ♂, 2 ♀), ♀ genitalia slide USNM 51385, J. Slotten. Georgia. Emanuel Co., Ohoopee Dunes Natural Area, Tract 1, wooded area, 0.3 mi N of Co. Rd. 160 (Hall’s Bridge Rd.) near Little Ohoopee River, 7 Sep. 2002 (1 ♂), J. Adams; Ohoopee Dunes N.A., 5 Oct. 2007 (2 ♂, 1 ♀), S.M. Lee, R.L. Brown. Tatinall Co., Ohoopee Dunes Area, 10 mi NE Lyons, Handy Kennedy Rd., 0.8 mi N of GA Hwy. 152, 23–25 Sep. 2009 (2 ♂, 2 ♀), J.K. Adams & I.L. Finkelstein. Mississippi. Jackson Co., Belle Fontaine Point, 14 Oct. 1998 (1 ♀). Paratypes deposited in CEH, CNC, MEM, and USNM.

**Figures 1–8.**
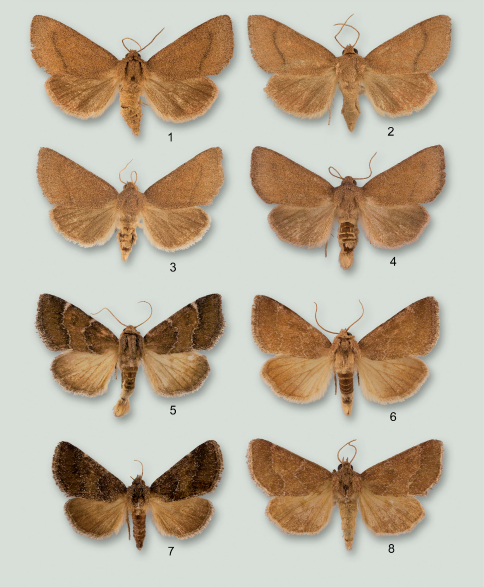
Adults of Schinia species. **1** Schinia psamathea, male holotype **2** Schinia psamathea, male paratype **3** Schinia psamathea, male paratype **4** Schinia psamathea, male paratype **5** Schinia saturata, male **6** Schinia saturata, male **7** Schinia saturata, female **8** Schinia saturata, male.

#### Etymology.

The specific epithet comes from the Greek noun, psamathos, for sand of the seashore. It is plural referring to the type of habitat that this species inhabits.

#### Diagnosis.

Eye large and globular; foretibia with a single, large, straight spinelike seta on inner apex; forewing ground color a dull medium brown; antemedial line absent; postmedial line black, slightly sinuate; hind wing pale rufous to gray; male abdominal sternites with well-developed hair pencils and pockets.

#### Description.

##### Male.

**Figures 9–12.**
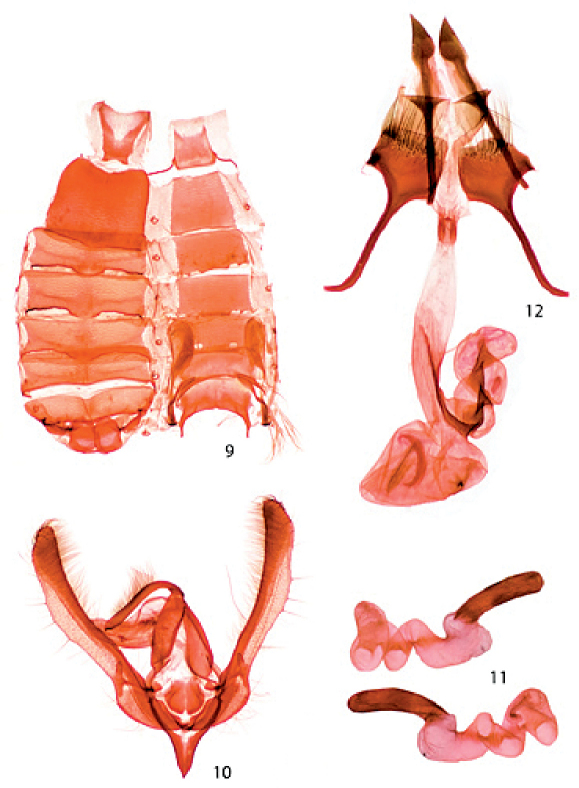
Abdomen and genitalia of Schinia psamathea. **9** Abdomen **10** Male genital capsule **11** Two lateral views of aedeagus **12** Female genitalia.

Head: Frons and vertex with light-brown scales tipped with white; labial palp curved, mostly white with light-brown and rufous scales; antenna filiform, scaled dorsally with white and brown; eye large and globular. Thorax: scales narrow, light brown tipped with white; foretibia a mixture of light-brown and white scales, inner margin with one large apical spinelike seta and from 1–3 progressively smaller spinelike setae, outer margin with 2–3 spinelike setae progressively smaller proximally, tarsi with light-brown and white scales, apical rings white; middle leg with light-brown and white scales; hind leg mostly white mixed with pale-rufous scales; underside white. Forewing (Figs. 1–4): Length 12.6–14.2 mm. From wing base to postmedial line scales a mixture of pale rufous and pale rufous tipped with rufous, giving a medium brown appearance; distal to the postmedial line scales are mostly pale rufous tipped with rufous giving a slightly lighter appearance than basal two-thirds; antemedial and medial lines absent; postmedial line slightly sinuate, dark brown; fringe a mixture of rufous and dark-brown scales tipped with white; underside pale rufous, central area darker, costal area and posterior margin lighter. Hind wing: pale rufous to gray; fringe white. Abdomen (Fig. 9): mostly cream colored with some scales tipped with pale rufous; hair pencils and scent pockets on sternites 2 and 4 well developed. Genitalia (Figs. 10–11): uncus moderately elongate, approximately 0.33–0.35 × length of valve; valve narrow, width approximately 8.3 × length, costal margin slightly curved, ventral margin curved, slightly produced at about 2/3 length of valve, and with a few stout setae along margin; apex of valve rounded; cucullus consists of a single row of less than 25 setae; ampulla wide, 0.05 × length of valve; juxta ovate, dorsal margin straight, lateral margins slightly flared; saccus narrow, V shaped; aedeagus slightly curved, apex produced to a dull point dorsally, minute dorsal scobinations from apex to approximately 0.3 × length; vesica with 2 coils.

##### Female.

As in male except forewing length 12.6–13.8 mm. Genitalia (Fig. 12): Papilla analis sclerotized, triangular, dorsal margin slightly concave, ventral margin greatly convex basally, apex pointed; ostium bursae consists of 2 slightly sclerotized lateral bars with a medial membranous area; ductus bursae elongate, membranous; appendix bursae with 2 coils, coiled sclerotized internal ribbon of appendix bursae extends approximately midlength into ductus bursae; corpus bursae ovate with produced apex; signa consists of 4 scobinate ribbons.

#### Distribution.

Schinia psamathea is known from east-central Georgia southwestward to the Panhandle of Florida, southeastern Alabama, and southwestern Mississippi.

#### Discussion.

Schinia psamathea is unique within the genus in having a simple forewing pattern that consists of only a slightly sinuate, dark-brown postmedial line and a solid-colored hind wing. Schinia saturata is a widespread species that occurs in Florida and somewhat resembles Schinia psamathea but is a dark-rufous moth compared to grayish-brown color of Schinia psamathea. The forewing markings will easily separate Schinia psamathea from Schinia saturata as the white antemedial line is usually present in Schinia saturata and the postmedial line is white in Schinia saturata and dark brown in Schinia psamathea. The inner margin of the foretibia has a single, large apical spinelike seta and 1–3 smaller proximal spinelike setae in Schinia psamathea and in Schinia saturata the large apical spinelike seta is more robust than in Schinia psamathea and there are 1–2 smaller proximal spinelike setae. The outer margin of the foretibia has 2–3 spinelike setae that get progressively smaller proximally in Schinia psamathea and in Schinia saturata there are 3–4 spinelike setae.

Both male and female genitalia are useful in separating Schinia psamathea from Schinia saturata. In the male genitalia, uncus is relatively longer in Schinia psamathea (0.33–0.35 × length of valve) than in Schinia saturata (0.28 × length of valve); valve is narrow (8.3 × length of valve) in Schinia psamathea and wide (6.7 × length of valve) in Schinia saturata; valve is only slightly angled just below apex in Schinia psamathea, but is abruptly angled at 3/4 length of valve in Schinia saturata; and corona consists of a single row of less than 25 setae in Schinia psamathea and contains five rows and more than 25 setae in Schinia saturata. In the female genitalia, papillae anales is triangular shaped in both species, but apex is sharply pointed and slightly curved in Schinia psamathea and in Schinia saturata apex is more rounded and not curved; on ninth segment the minute spicules are short in Schinia psamathea and long in Schinia saturata; and on eighth segment the distal setae are elongate (extend to or beyond the distal margin of the posterior apophyses) and dense in Schinia psamathea, whereas in Schinia saturata the distal setae are short and sparse. Shared characters between Schinia psamathea and Schinia saturata include the abdominal hair pencils and associated pockets, two coils in the male vesica, two coils in the female accessory bursae, and triangular shaped papillae anales.

There is some variation in the forewing pattern of Schinia psamathea. The postmedial line varies from being quite visible to almost absent. The hind wing is usually gray, but in some individuals it can be pale rufous.

Schinia psamathea seems to prefer sandy soils either in dune type habitats or near sandy beaches. Moths are active in the mid- to late afternoon and are attracted to light. They nectar on several different flowers and the flight is similar to other day-flying Schinia, being very fast and darting. Adults fly between 7 September and the end of October, being most abundant in mid-October (J.K. Adams, pers. comm.).

A possible host plant for Schinia psamathea is woody goldenrod (Chrysoma pauciflosculosa (Michx.) Greene, Asteraceae), which is a small sprawling, evergreen shrub with thick, almost succulent, grayish-green leaves and bright yellow flowers that bloom in late summer. Woody goldenrod occurs in sandy scrub and sandhills habitats along the Fall Line in the Carolinas and Georgia, and on the Coastal Plain in Alabama, Mississippi, and the Florida Panhandle. Along the Gulf Coast, woody goldenrod occurs in coastal scrub behind the primary dune system. Other plant associations in the sandhills include turkey oak (Quercus laevis Walter, Fagaceae) and longleaf pine (Pinus palustris Mill., Pinaceae). In scrub habitats the woody goldenrod is associated with sand live oak (Quercus geminata Small, Fagaceae) and sand pine (Pinus clausa (Chapm. Ex Engelm.) Vasey ex Sarg., Pinaceae) (Chritsman 2008).

The known distribution of Schinia psamathea in Mississippi, Alabama, Florida, and Georgia completely overlaps the distribution of woody goldenrod. The state and county distribution of woody goldenrod from west to east is given here as a guide to further explore the distribution of Schinia psamathea: Mississippi (Harrison and Jackson Counties), Alabama (Mobile and Baldwin Counties), Florida (Escambia, Santa Rosa, Okaloosa, Walton, Washington, Bay, Calhoun, Gulf, Liberty, Franklin, and Wakulla Counties), Georgia (Wheeler, Emanuel, and Tatinall Counties), South Carolina (Lexington County), and North Carolina (Robeson County) (Wunderlin and Hansen 2008).

## Supplementary Material

XML Treatment for Schinia psamathea
